# Poliovirus eradication initiatives in dire straits: Updates from Pakistan

**DOI:** 10.7189/jogh.11.03036

**Published:** 2021-03-01

**Authors:** Nafhat Shaikh, Noor Fatima, Syed Zaki Muhammad

**Affiliations:** 1 Department of Internal Medicine, Liaquat University of Medical and Health Sciences, Jamshoro, Pakistan; 2 Department of Internal Medicine, Liaquat National Hospital and Medical College, Karachi, Pakistan; 3 Department of Internal Medicine, Dow University of Health Sciences, Karachi, Pakistan

Early in the 20^th^ century, polio epidemics in industrialized nations made it the most feared disease, paralyzing countless children. In the mid of the 20^th^ century, introduction of effective vaccines reduced the number of polio cases from the developed countries, however, many developing countries continued to experiences unrestrained transmission of the virus. In 1988, the Global Polio Eradication Initiative (GPEI) was launched by the World Health Assembly with the aim to eradicate polio from the whole world. This program was a global collaborative effort, headed by national governments, WHO, UNICEF and other agencies. Billions of children have been immunized and the number of wild polio virus (WPV) cases has declined by 99%, since then [1,2].

The strategic development of the program with resource mobilization and regular immunization drives eradicated polio from American, western Pacific and the European region by the year 2002 [3] and the entire South-East Asian region in 2014 after India thrived to eradicate polio for good [4]. The latest success story came in 2020, when the African region was declared WPV-free, when Nigeria, one of the last three countries to harbor the virus, wiped out WPV in a historic milestone [5]. Two out of 3 strains of the WPV have also been successfully eradicated after the last reported case of type 2 WPV in 1999 and type 3 WPV in 2012. This was made possible by the collaboration of 200+ countries, millions of volunteers, and huge sums of investment.

Tackling the last percent of WPV cases continues to pose a challenge in Pakistan and Afghanistan where poor infrastructure, hard to reach populations, political instability, and conflict hamper the efforts to contain the virus. Pakistan took polio eradication initiative in 1994 which went quite well until the program took a hit when the drone attacks in federally administered tribal areas and militant groups hampered the vaccination campaigns [6]. Following years did show a bright spot when the incidence rate significantly decreased.

However, once again the resurgence of poliovirus infections has put Pakistan in a daunting epidemiological situation. After successfully reducing the tally to 8 cases in 2017, the number of WPV cases has only risen since then with 12 cases in 2018 and 149 cases in 2019. The independent monitoring board of the GPEI in 2019, were informed of the WPV outbreaks befalling polio-free regions of Pakistan alongside the traditional core reservoirs. This trend continued in 2020, where circulation of the virus was observed not only in known reservoirs of Karachi, southern districts of Khyber Pakhtunkhwa, and in districts of Balochistan, but also in other less affected areas of Sindh and Punjab provinces [7]. According to updates by the Pakistan Polio Eradication Program, till November 2020 environmental surveillance reported 386 samples positive for WPV, a 21% increase from 319 positive samples in 2019. The total number of WPV cases reported in 2020 were 84; highest number of cases were from Baluchistan province at 26, followed by 22 cases each in Sindh and Khyber Pakhtunkhwa, and 14 cases in Punjab. Although this is a significant reduction in cases compared to 2019, this could well be an underrepresentation owing to the coronavirus disease pandemic restrictions implemented in the country [2].

The escalation of this crisis is an outcome of many factors including misconceptions about the vaccine, mistrust amongst communities, subpar performance of officers, and political disunity. Vaccination programs are particularly not well-received in communities deprived of the basic infrastructure, such as access to quality education, clean water supply, and proper sanitation, which causes resentment. The authorities have failed to educate people regarding the need for multiple vaccine doses and eliminate the suspicions about the vaccine being injurious, resulting in high dropout rates [8]. Besides, programmatic inefficacies including lack of accountability and deficiently integrated strategies add to the damage. Polio eradication is seen as a foreign program by some communities and this is another factor that contributes to the problem. This is due to limited representation of prominent local figures in leadership positions, and the fact that major technical and strategic support is provided by foreign agencies, which sometimes overlook local input. [9]. Moreover, the coronavirus disease (COVID-19) pandemic has had a significant impact on the polio programs after their workforce and resources were deployed to counter the pandemic, and mass immunization campaigns were suspended to mitigate its spread [10].

**Figure:**
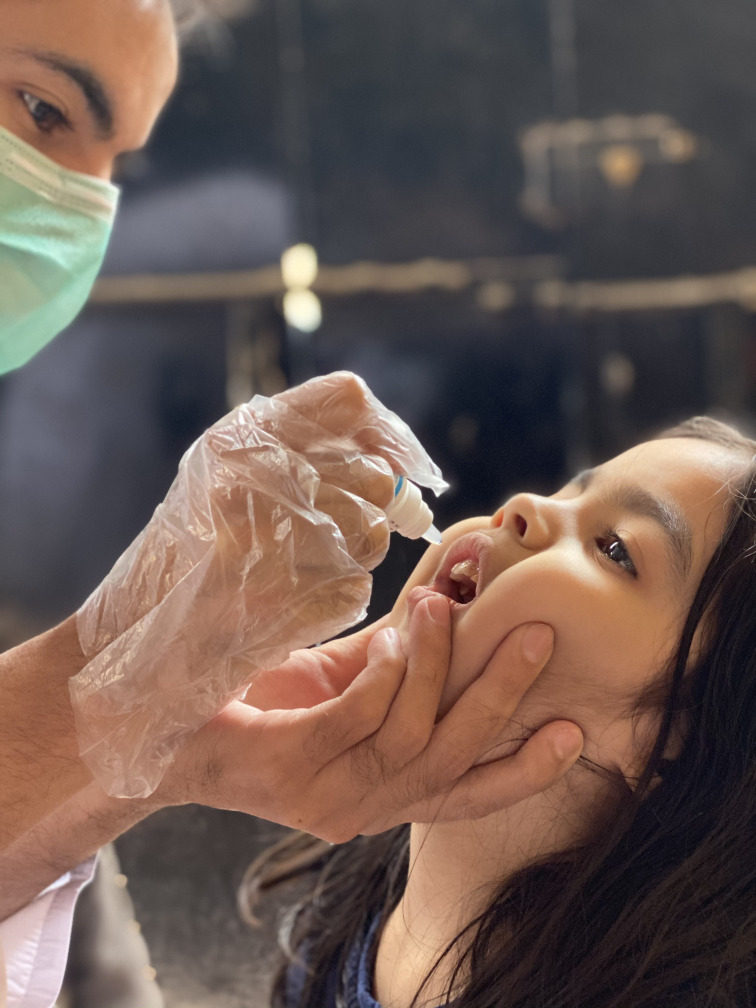
Photo: from Dr Nafhat Shaikh’s collection, used with permission.

Therefore, the national framework towards polio eradication must be restructured. This would require political synergy at both the national and provincial levels. The government needs to ameliorate primary health conditions with clean water and proper sanitation which would not only hinder proliferation of the virus but improve trust and acceptance of its programs. Increasing local representation can bring local input to the discussion, and involving community leaders could help devise community-specific solutions. These local partnerships would help structure the plans meaningfully. The COVID-19 pandemic response by the government of Pakistan, is an example of how we could confront public health issues emergently, if there is political unity and seriousness. This silver lining during the pandemic is evidence that we could wipe out WPV from Pakistan if the government takes ownership, brings all parties together, and manages Polio infections with the seriousness required for a national emergency.

Research is an extremely significant part of resolving public health issues and should be utilized to inform innovation and development. Likewise, to enter a polio-free world we must continue to gather evidence that will guide us through. In addition to technical epidemiological data, collection and utilization of social data to devise specific strategies could possibly address issues of vaccine refusal. Furthermore, over the recent years similar models of vaccine delivery have been used and have resulted in little progress towards the finish line. It is therefore imperative to test alternative delivery models to evaluate their effectiveness. One such model could be the integration of polio vaccines in essential childhood immunization program. It is essential now for the polio programs to stop looking back at past glories and adapt to the circumstances of today, because the world is transforming at an unbelievable pace and the strategies that helped the polio program few years back, may not work at present.
